# Lupus

**Published:** 1874-05-15

**Authors:** E. Andrews

**Affiliations:** Mercy Hospital


### LUPUS. — FISTULA IN ANO.

SUBSTANCE OF A CLINIC BY PROF. E. ANDREWS, M.D., IN MERCY HOSPITAL, MAY 4TH, 1874. REPORTED BY J. R. KEWLEY.

GENTLEMEN: To-day we have, as you see, a patient afflicted with lupus. We propose to cure him, if possible, by an operation. This disease is one whose exact nature is not very well understood by pathologists, owing to the fact, I think, that the term "lupus" is applied to really different diseases. True lupus strongly resembles cancer (after ulceration), both to the unaided eye, and by the examination of the morbid tissues by the microscope. However, if a cancerous ulcer exists, the surrounding lymphatics are generally enlarged. Not so with lupus: the lymphatics remaing normal and healthy.

Lupus begins as a small ulcer, with a thickened, ragged edge, rapidly spreading. In this case, we see it has encroached upon the nose, upper lip, and quite a large portion of the cheek, and has partially destroyed the left superior maxillary bone. Local treatment must be pursued, constitutional measures being wholly insufficient in all these cases. The ulcer is sometimes cauterized. In London I saw it so treated, with a red-hot iron, by the surgeons of that city.

Some German surgeons recommend the scraping away of all the morbid tissue that is possible, by means of some blunt instrument, and then hacking with numerous minute scarifications, and, after this operation, cauterizing the parts with fuming nitric acid. This stops the spreading of the disease; and soon it is superseded by a healthy ulcer, with healthy granulations, which goes on until our labors are crowned by seeing a perfect cicatrix. Sometimes, however, such is not the case, and then the operation must be repeated. In this operation I always try and remove all the morbid parts, although the ulcer's center may not present a lupoid character..

The patient, an elderly gentleman, being now fully under the influence of sulphuric ether, the Professor proceeded to operate according to the German method described above, removing, during the operation, some small spiculæ of bone from the superior maxillary. The disease, as was feared, extended somewhat into the air-passages.

Gentlemen: I will now call your attention to this colored man, of middle-age, suffering from fistula in ano. These fistulæ are generally caused by a small abscess in the areolar tissue around the rectum. This abscess first breaks into the bowel, and afterwards, working its way through the tissues, opens upon the external surface, thus producing a communication between the bowel and the outer world, different from that which nature decreed. The opening thus established is prevented from healing by the irritation of foreign material seeking to find an exit through it. Some of our medical brethren object to an operation on fistulous patients of consumptive tendencies, on the ground that, after the operation, the tubercular disease is more rapidly developed. This opinion is not conclusively proven, by any means; yet there may be truth in it; and I would not operate on consumptive patients.

In this patient, as you see, there is a perfect net-work of external openings. By introducing a probe into this large opening on the left side of the anus, and a finger into the rectum, I can ascertain the locality of the internal opening into the bowel. Also on the right side, where we have a large fistula. In this one I find that, instead of opening directly into the rectum (as the one on the left side did), the probe runs behind the anus, into the fistulæ of the opposite side.

Introducing a grooved director into the larger of the fistulæ of the left side, as a guide to my bistoury, I cut through the tissues and the walls of the rectum into that organ, not sparing the sphincter muscles. Now, on the right side, as the principal fistula does not open into the rectum, we will introduce the bistoury on the grooved director, and cut down behind the rectum, terminating in the incision of the left side, thus severing the sphincter muscles but once, which will add much to their future strength. You will observe that the incisions on the opposite sides are made in such a manner that if any cicatricial contraction takes place in healing, the anus will not be held open by the traction of two cicatrices in opposite directions, as might be the case if I cut the sphincters through upon both sides.

Now, we will open some of these smaller fistulæ into the main and connected incisions already made, and afterwards prevent union by first intention, by introducing into the incision some lint covered with cerate. We expect to see healthy granulations appear in the bottom of these incisions, which will continue to develop from the bottom in this way until a complete cicatrice is formed.

